# Tracking Tumor Colonization *in* Xenograft Mouse Models Using Accelerator Mass Spectrometry

**DOI:** 10.1038/s41598-018-33368-0

**Published:** 2018-10-09

**Authors:** Nicholas R. Hum, Kelly A. Martin, Michael A. Malfatti, Kurt Haack, Bruce A. Buchholz, Gabriela G. Loots

**Affiliations:** 1 Lawrence Livermore National Laboratory, Physical and Life Sciences Directorate, Livermore, CA USA; 2 Georgetown University, Department of Biochemistry & Molecular Biology, Washington, DC USA; 3 Center for Accelerator Mass Spectrometry, Lawrence Livermore National Laboratory, Livermore, CA USA; 4 UC Merced, School of Natural Sciences, Merced, CA USA

## Abstract

Here we introduce an Accelerator Mass Spectrometry (AMS)-based high precision method for quantifying the number of cancer cells that initiate metastatic tumors, in xenograft mice. Quantification of ^14^C per cell prior to injection into animals, and quantification of ^14^C in whole organs allows us to extrapolate the number of cancer cells available to initiate metastatic tumors. The ^14^C labeling was optimized such that 1 cancer cell was detected among 1 million normal cells. We show that ~1–5% of human cancer cells injected into immunodeficient mice form subcutaneous tumors, and even fewer cells initiate metastatic tumors. Comparisons of metastatic site colonization between a highly metastatic (PC3) and a non-metastatic (LnCap) cell line showed that PC3 cells colonize target tissues in greater quantities at 2 weeks post-delivery, and by 12 weeks post-delivery no ^14^C was detected in LnCap xenografts, suggesting that all metastatic cells were cleared. The ^14^C-signal correlated with the presence and the severity of metastatic tumors. AMS measurements of ^14^C-labeled cells provides a highly-sensitive, quantitative assay to experimentally evaluate metastasis and colonization of target tissues in xenograft mouse models. This approach can potentially be used to evaluate tumor aggressiveness and assist in making informed decisions regarding treatment.

## Introduction

Currently, ~1.6 million (M) new cases of cancer get diagnosed annually, and the American Cancer Society has estimated that over 0.5M cancer patients will die in the US this year alone. While a large fraction of primary tumors can be treated if detected early, metastatic cancer is generally incurable and accounts for the majority of cancer-related deaths. Virtually all cancers can metastasize and common metastatic sites include bone, liver and lung. Understanding the molecular and biological basis of metastasis is essential for conquering it, however, there are very few precise tools that allow us to study the process of metastasis. In particular, we lack highly sensitive methods to quantify metastatic tumor burden in experimental models.

Over the last few decades, rodent models have significantly contributed to our current knowledge of cancer. They have been used as proxies for humans for (1) discovering and testing new therapies to improve cancer outcomes, (2) finding better ways to detect cancers at early stages when malignancies are most curable, (3) assessing new approaches to cancer prevention and (4) for determining genetic risk factors of developing cancer, therapeutic responsiveness, and therapy-induced toxicity^1^. Non-invasive imaging techniques, including magnetic resonance imaging (MRI) and computed tomography (CT) have also been adapted to small laboratory animals to better study cancer metastasis *in vivo*^2^. While these methods have been insightful, they fail to provide an understanding of metastasis at the single cell level.

Currently, there are a variety of technologies used to modify cancer cells to better study them *in vivo*. One method utilizes bio-luminescence to track genetically altered cancer cells expressing firefly luciferase (*luc*) and has been useful in studying metastasis stemming from intra-cardiac (IC); intra-osseous (IO), or arterial tail vein (TV) delivered *luc*^+^ tumor cells. *In vivo* implantation or systemic injection of tumor cells transfected or transduced with *luc* allows monitoring of tumor growth and migration by measuring the photon signals emitted throughout the animal’s body. As the cells migrate and lodge onto different organs, their location and expansion can be tracked by luminescence^3,4^. This technology has helped derive new insights into many types of cancers including but not limited to: pheochromocytoma^5^, breast cancer^6^, osteosarcoma^7^, prostate cancer^8^, mesothelioma^9^, as well as helped assess therapeutic potential of single or co-administered drugs in xenograft animal models^10–14^. While this approach has broad applications, it poses several limitations: (1) cancer cells must be genetically modified to introduce the *luc* reporter gene; (2) *luc* signal is dependent on *luc* gene expression, therefore it is susceptible to micro-environmental changes in the organism that may affect the transcription level of the reporter gene; (3) measurements are not truly quantitative since tumor size and location is extrapolated based on luminescence intensity, and high intensity focal signal may ‘spill’ into adjacent tissues making it difficult to delineate tumor boundaries or exact visceral location^15–17^.

A different *in vivo* labeling method takes advantage of the highly proliferative characteristic of tumor cells through the administration of [^18^F]-fluoro-3′-deoxy-3′-L-fluorothymidine ([^18^F]FLT) and measures cancer proliferation using positron emission tomography (PET). [^18^F]FLT is taken up by all cells, but actively dividing cells such as cancer cells phosphorylate [^18^F]FLT to generate [^18^F]FLT-monophosphate; [^18^F]FLT-monophosphate becomes trapped intracellularly and marks actively dividing cells^18^. Unlike radioactively labeled thymidine (^14^C-thymidine) that has been shown to robustly incorporate into newly synthesized DNA, only 0.2% of administered [^18^F]FLT incorporates into cellular DNA, *in vitro*, because it acts as a chain terminator^18^, hence [^18^F]FLT uptake correlates with the first step in the salvage pathway of DNA synthesis. One application of this technique has been to measure viability and tumor growth during or after treatment with anticancer agents. While studies using mouse xenografts have shown that [^18^F]FLT *in vivo* imaging can be used to monitor the effects of cancer therapy^18,19^, its utility is limited for a few reasons. Mainly, label uptake is nonspecific, and can sometimes mark metabolically active non-cancer cells leading to false positive scans^18^. Additionally, the short half-life (20 min) of ^18^F precludes analyses over long periods of time, limiting the type of experiments and biological questions that can be addressed *via* this method.

Recently, researchers have introduced microparticle-based materials detectable via PET imaging with the eventual goal of delivering therapeutics in a targeted manner. Starch-based microparticles (~30 μm average diameter) have been functionalized with radioactive ligands such as Gallium-68 and Rhenium-188 *via* an amino linker^20^. The specificity of microparticles combined with conjugated radiolabels detectable *via* PET imaging has provided a new avenue to detect cancer cells *in vivo*. While microparticles radiolabeled with Gallium-68 are an improvement over luminescence assays, this technique still lacks single cell resolution and is not a direct method for quantifying single cells.

Single cell quantification using ^14^C-thymidine that has been directly incorporated into a cell’s DNA provides a new and exciting opportunity for assessing tumor burden and metastatic potential. Accelerator Mass Spectrometry (AMS) is used to detect and accurately quantify attomolar concentrations of radioisotopes directly isolated from cancer cells and organs of interest^21^. This technology has also been used to evaluate adduct formation and drug resistance in bladder cancer in addition to investigating pharmacokinetics of candidate drug compounds^22,23^. Unlike other methods where incorporation of [^18^F]FLT is dependent on rate of proliferation not specific to cancer cells or bio-luminescence assays where detection is not precise, ^14^C-thymidine is only introduced through cancer cells that have been labeled and quantified *ex vivo* via AMS, allowing the investigation of tissue colonization and metastasis.

While comprehensively the data collected from animal models has greatly improved our understanding of cancer metastasis, the limitations for detecting metastatic tumors in humans have persisted in rodents, and currently no quantitative method exists that can accurately determine the presence of a single metastatic cancer cell or the complete eradication of it in response to therapy. Here we introduce an AMS-based approach that allows us to quantitatively assess the metastatic potential of ^14^C-thymidine labeled cancer cells, in rodent xenograft models. Since cancer is a highly heterogenous disease, this method can now be further developed to study patient derived tumor xenograft models (PDTX) where patient tumors can be expanded *in vivo*, and used in drug efficacy studies.

## Results

### ^14^C-thymidine Labeling Allows for Single Cell Detection of Colonized Tumor Cells by AMS

**Figure 1:**
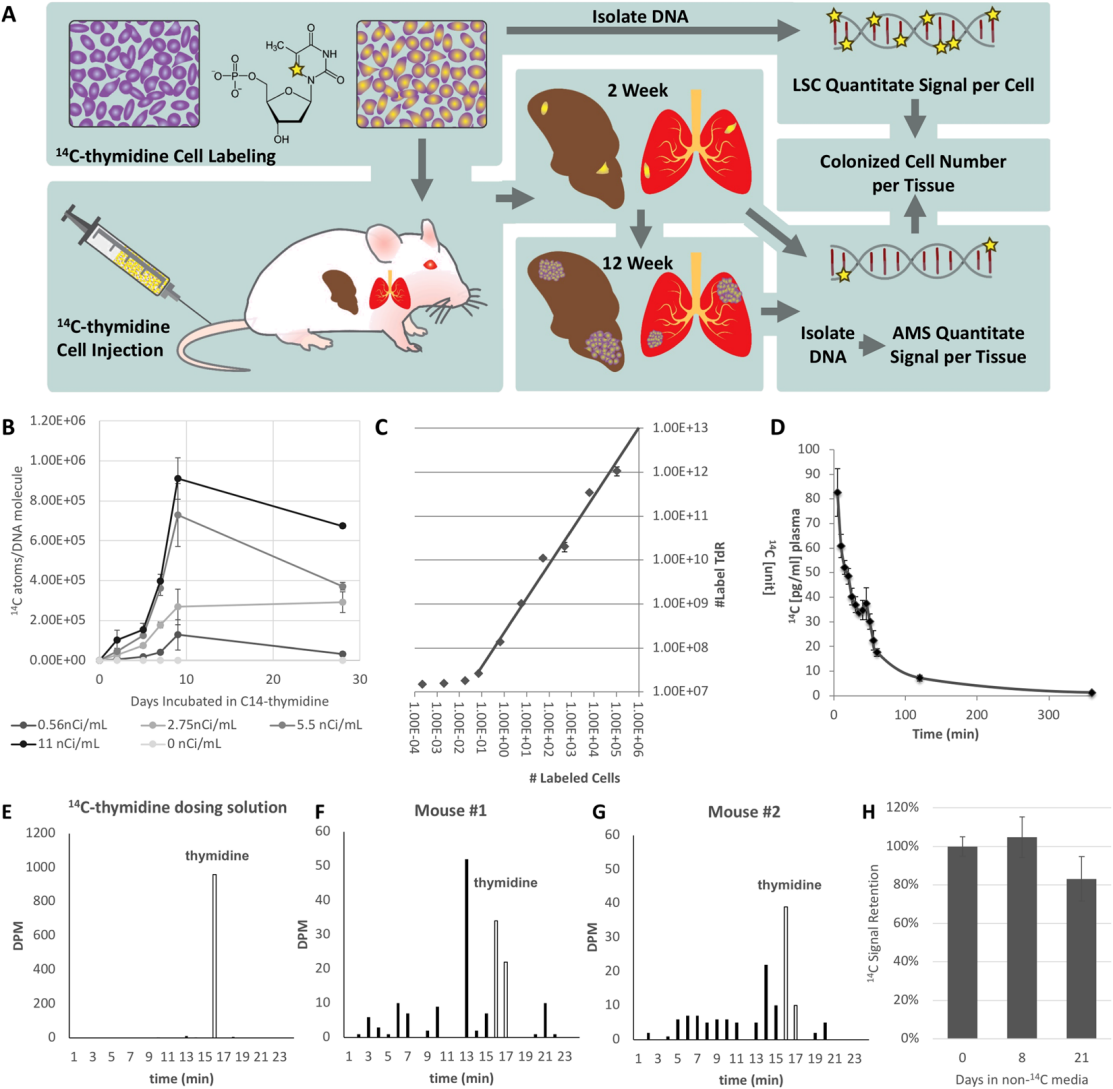
Workflow and validation of ^14^C-labeling cancer colonization assay. (**A**) Schematic of colonization assay workflow. Cells were first cultured with ^14^C-thymidine media to achieve single cell resolution and injected into NSG mice *via* tail vein (TV), heart (IC) or subcutaneous (SQ) routes of delivery. Injected cells were allowed to metastasize for up to 12 weeks. Tissues were harvested at early (2 weeks post injection) and late (12 weeks post injection) time points and DNA was isolated and quantified using AMS. In parallel, the activity of ^14^C-thymidine label in cultured cells was quantified using liquid scintillation counting (LSC). AMS measurements and LSC readings were combined to calculate the number of colonized cells per each organ examined. (**B**) Optimization of ^14^C-thymidine labeling *in vitro* using various concentrations of radioactivity in cell culture media (n = 3). (**C**) Correlation of experimental versus theoretical values of ^14^C-thymidine in labeled cells. (**D**) Pharmacokinetics of free ^14^C-thymidine (n = 6): mean concentration-time profile of thymidine in blood following a single intravenous administration of 700 pCi/animal in C57Bl/6 male mice. Data is expressed as the average per time point ± standard error. (**E**) ^14^C-thymidine dosing solution administered to C57Bl/6 male mice, thymidine retention time = 16 min. (**F**,**G**) HPLC chromatographs of mouse urine collected at 24 h post dose from 2 mice exposed to ^14^C-thymidine. Open bars indicate thymidine retention time. (**H**) Stability of ^14^C-thymidine in labeled cells transferred to non-^14^C-thymidine media.

Quantitation of colonized tumor cells at sites of metastasis was accomplished by measuring the ^14^C signal in DNA of various tissues of animals injected with cancer cells whose DNA was labeled with ^14^C-thymidine. Organs with or without visible tumors were harvested and the DNA from a fraction of the homogenized sample was quantified by AMS to determine the ^14^C signal^24,25^. ^14^C tissue level was converted to quantities of colonized cells based on the ^14^C levels detected in labeled cells prior to delivery into the animal. This approach allowed us to ascertain whether any of the injected cells colonized distant organs as depicted in Fig. 1A.

Based on typical sensitivity and setting the limit of quantitation (LOQ) as the average of undosed controls plus 2–3x the standard deviation^24,26,27^, we estimated that incorporation of 1 × 10^6 14^C-thymidines into one mammalian genome [~6.2 pg of total DNA/genome] would enable single cell detection by AMS. The DNA of PC3 cells cultured in the presence of ^14^C-thymidine at 4 different specific activities [0.55–11 nCi/mL] was examined for ^14^C-thymidine levels at days 2, 5, 7, 9, and 14. A specific activity of 11 nCi/mL was sufficient to incorporate 1 × 10^6 14^C-thymidine/cell over 7 days of culture (Fig. 1B). Next we compared the theoretical ^14^C-values to the experimental values using AMS from a single cell measurement by analyzing the equivalent DNA from 1 × 10^6^ cells spiked with an exponential serial dilution of ^14^C-labeled DNA representing differing quantities of founder cells [1:1,000,000; 1:100,000; 1:10,000; 1:1,000]. Extrapolated cell quantities from AMS signal yielded a linear relationship relative to estimated ^14^C-labeled cells down to the equivalent of 0.1 labeled cells/10^6^ total cells (Fig. 1C), suggesting that this method could allow the detection of 1 labeled cell in 1 × 10^7^ unlabeled cells. All proceeding experiments described herein were conducted with cells labeled by culturing with ^14^C-thymidine at a specific activity of 11 nCi/ml over 7 days.

Since thymidine is readily incorporated during S phase by mitotic cells, measured ^14^C in DNA should accurately correlate with the presence of tumor cells *via* presence of ^14^C in newly synthesized DNA^28^. We quantified the pharmacokinetics of ^14^C-thymidine over a 24 hour period at physiological levels to ensure that recycled ^14^C-thymidine is not incorporated into healthy cells. The plasma concentration *vs*. time curve followed first order kinetics with a distribution half-life of 0.46 h and an elimination half-life of 10.5 h indicating rapid distribution beyond the plasma compartment. Clearance was also rapid at 1.03 L/h/kg. No measurable ^14^C-thymidine was detected in the plasma after 6 hours post injection (Fig. 1D). In addition, HPLC separation and LSC analysis of urine collected between 0 and 24 h post dose from mice intravenously exposed to ^14^C-thymidine revealed that ~2.45% of total ^14^C-thymidine was present intact, in the urine. This data suggests that ^14^C-thymidine is rapidly cleared and therefore ^14^C-thymidine released metabolically from apoptotic cancer cells is unlikely to be recycled and incorporated into actively dividing host cells (Fig. 1F,G; Table 1).

**Table 1:** Percent of ^14^C radioactivity recovered in 24 h urine after a single IV dose of ^14^C-thymidine in male *C57Bl/6* mice.

	% of total ^14^C dose recovered in urine	% of ^14^C dose recovered as thymidine in urine
mouse 1	8.0	2.6
mouse 2	6.5	2.3

To further examine ^14^C-thymidine signal stability in rapidly dividing cancer cells, labeled PC3 cells (prostatic carcinoma) were incubated in non-radioactive media to simulate growth for prolonged durations. Total input ^14^C activity was measured at the time cells were seeded, and total signal retention was calculated over time, in expanded cultures. No detectable signal was lost from the population 8-days post seeding. A 17% signal loss was measured at 21-days post seeding however, this loss was not significant when compared to samples collected at the time of cell seeding (*p*-value = 0.0829) (Fig. 1H). Therefore, we conclude that ^14^C activity is retained and stable within cell populations over long term culturing and any ^14^C-thymidine detected in the tissues of xenograft mice is derived from the originally injected cancer cells and their progeny, only.

### Tumor Cell Colonization in Xenograft mice is cell line specific

**Figure 2:**
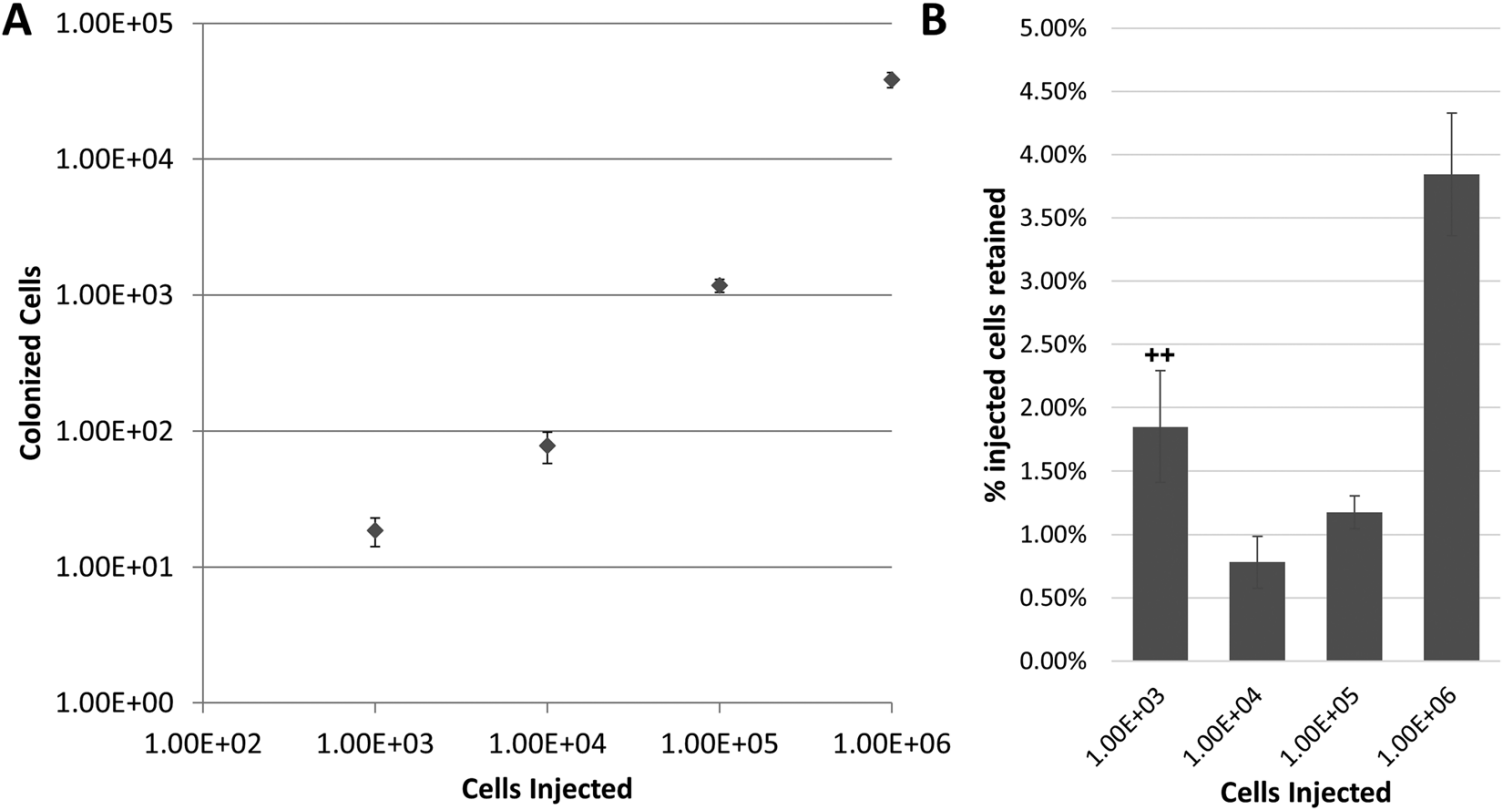
Quantification of subcutaneous tumor forming cells. Comparison of subcutaneously injected cells in relation to the number of colonized cells quantified using AMS (**A**) from the harvested tumor. Percentage of initially injected ^14^C-labeled cells retained in final tumor (**B**). At least 4 replicates were analyzed for each quantity of cells injected with the exception of 1.00E + 03 cells due to ^14^C signal below the level of detection (++).

To determine the persistence of ^14^C-signal in growing tumors, we injected a logarithmic range of PC3 labeled cells subcutaneously (SQ) [1 × 10^3^–1 × 10^6^], allowed tumors to reach the same size [1 cm^3^], then quantified the total amount of detectable ^14^C in only the excised SQ tumors. Interestingly, very few injected cells initiated the tumor regardless of the quantity of cells delivered (Fig. 2A). Tumors originating from larger quantities of injected cells yielded greater rates of colonization, where 1 × 10^6^ injected cells retained ~ 3.84%, 1 × 10^5^ injected cells retained ~1.18% and 1 × 10^4^ injected cells retained ~0.78% of the total delivered cells. Tumors initiated from 1 × 10^3^ cells retained less than 1.85% in the final tumor, however the ^14^C levels were below the LOD in 2/4 analyzed tumors (Fig. 2B). This data suggests that >95% of the injected human cancer cells delivered into NSG mice SQ die, and that the emerging SQ tumor is initiated by a very small population of tumor initiating cells.

**Figure 3:**
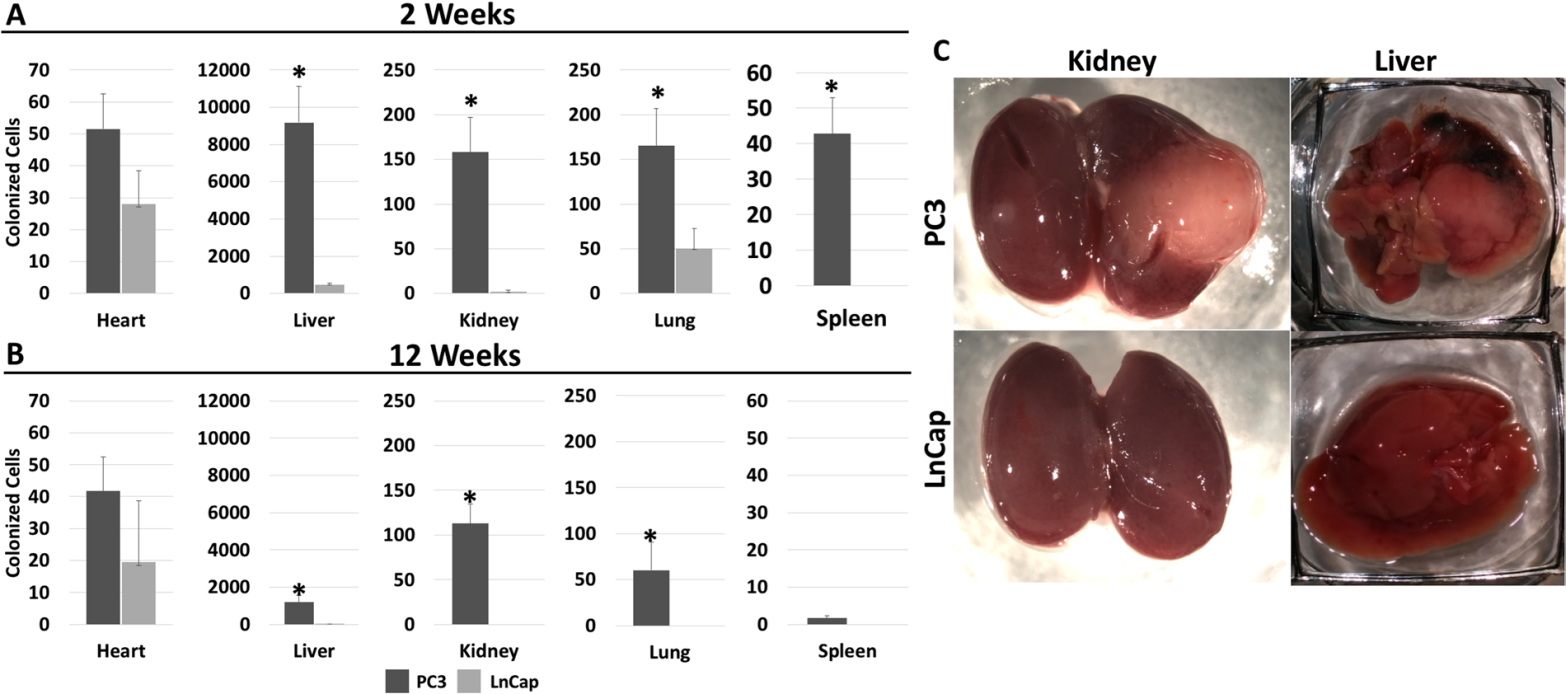
Colonization of tissue from PC3 and LnCap cells. Quantity of colonized founder cells calculated from ^14^C signal in DNA from target tissues isolated (**A**) 2 weeks post injection (n ≥ 5) and (**B**) 12 weeks post injection (n ≥ 4). Error bars represent SEM. *p < 0.05. (**C**) Representative pictures of kidneys and livers from PC3 or LnCap injected animals.

To determine the dynamics of tissue colonization in PDTX, we label PC3 [highly metastatic] and LnCap [non-metastatic]^29^ prostate cancer cell lines with ^14^C-thymidine at single cell resolution and analyzed their respective patterns of metastasis post intravenous delivery into NSG mice. Previously reported metastatic sites for PC3 cells [lung, liver, kidney] and non-metastatic sites [heart, spleens]^30^ were harvested at 2- and 12- weeks post injection, independent of the presence of visible tumors. ^14^C signal was converted to colonized cell quantity based off the ^14^C activity per injected cell. Additionally, uninjected control tissue background signal was subtracted prior to quantification. At 2 weeks post-delivery, ^14^C signal was detected in the DNA of tissues harvested from both PC3 and LnCap lines and the signal was present in both metastatic and non-metastatic tissues (Fig. 3A). The highest signal for both cell lines was observed in the liver, however PC3 derived tissues had significantly higher levels in all tissues examined, except heart, suggesting that tumor cells colonize a wide range of tissues, independent of the sites previously determined to bear secondary tumors. Although significantly lower, LnCap cells also colonized target tissues at 2-weeks post injection, suggesting that TV delivered tumor cells reach distant sites at very low frequency but can reside in these tissues for up to 2 weeks without initiating tumors.

At 12-weeks post injection, the PC3 injected mice had visible tumors in all previously reported organs with the most striking tumors visible in the liver and kidney (Fig. 3C). In contrast, the LnCap mice had healthy organs with no visible tumors. ^14^C quantification in these tissues revealed that consistent with the presence of tumors, PC3 organs had significantly higher levels of ^14^C while the LnCap organs had cleared most of the ^14^C detected at the 2-week time point. These data suggest that most ^14^C-labeled LnCap cells injected into the mice have cleared from these organs, while the PC3 cells proceeded to form aggressive tumors (Fig. 3B). ^14^C signal from PC3 injected animals in all target tissues was decreased at the 12-week time point relative to the 2-week time point in all tissues, except for heart, a non-metastatic site, suggesting that the heart may be a site for dormant tumor cells. Despite the attenuation of signal from 2- to 12-weeks, the colonized cell profile produced *via* this assay recapitulated visible tumor formation for both cell lines at both an early and late time point.

### Tissue Colonization is Dependent on the Route of Tumor Delivery

Several studies have observed altered metastatic profiles based on the route of cancer cell introduction^31,32^. Labeled PC3 cells were delivered *via* TV or IC and tissues were harvested prior to the formation of any visible tumors (2-weeks post injection) and at later time points when tumors were visible (7-weeks post injection for IC and 12-week post injection for TV) to compare the two delivery routes. TV delivered PC3 cells produced lung and kidneys tumors in contrast to IC delivered cells which yielded heart, lung, liver, and kidney tumors upon visual inspection at time of tissue harvesting (Table 2). Also, IC delivery led to more rapid tumorigenesis, such that kidney and liver tumors developed by 7-weeks post injection were similar in size to those developed after 12-weeks post TV injection.

**Table 2:** Percent of animals with visible tumors.

	Heart	Lung	Liver	Kidney
IC	100%	100%	67%	17%
IV	0%	33%	100%	83%

**Figure 4:**
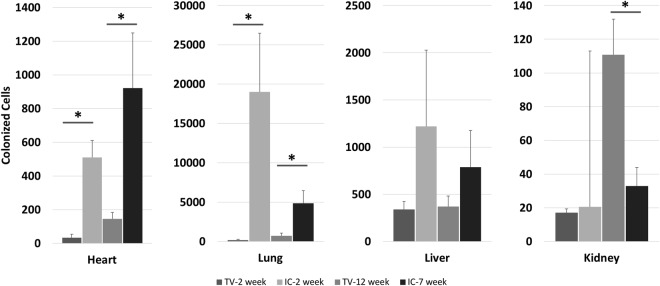
Tail vein and intracardiac injected cancer cell colonization. Profile of colonized cells in target tissues calculated from ^14^C signal in DNA from target tissues isolated at 2-week post injection, 7-week for intracardiac (IC), or 12-week for tail vein (TV) (n = 5).

Furthermore, unique profiles of dispersion of colonized cells was evident at both early and late time points with statistically significant quantities of colonized cells differentiated by route of injection found in the heart, lung, and kidney (Fig. 4A–D). These distinct metastatic profiles of cell colonization were consistent with observed metastases found at both 2-week and 12-week time points. IC injected animals contained colonized cells in heart, lung, liver, and kidney. Cells injected IC preferentially targeted the lung at both time points followed by heart and liver colonization containing equivalent quantities of ^14^C labeled cells. Moreover, kidney ^14^C signal was low in both early and late time points, consistent with the lower rate of metastasis observed at 7 weeks post injection. TV injected animals resulted in lower total quantities of colonized cells in target tissues which is consistent with the increased tumorigenicity observed in IC compared to TV administered animals.

### Cancer cell colonization correlates to tumorigenic activity

**Figure 5:**
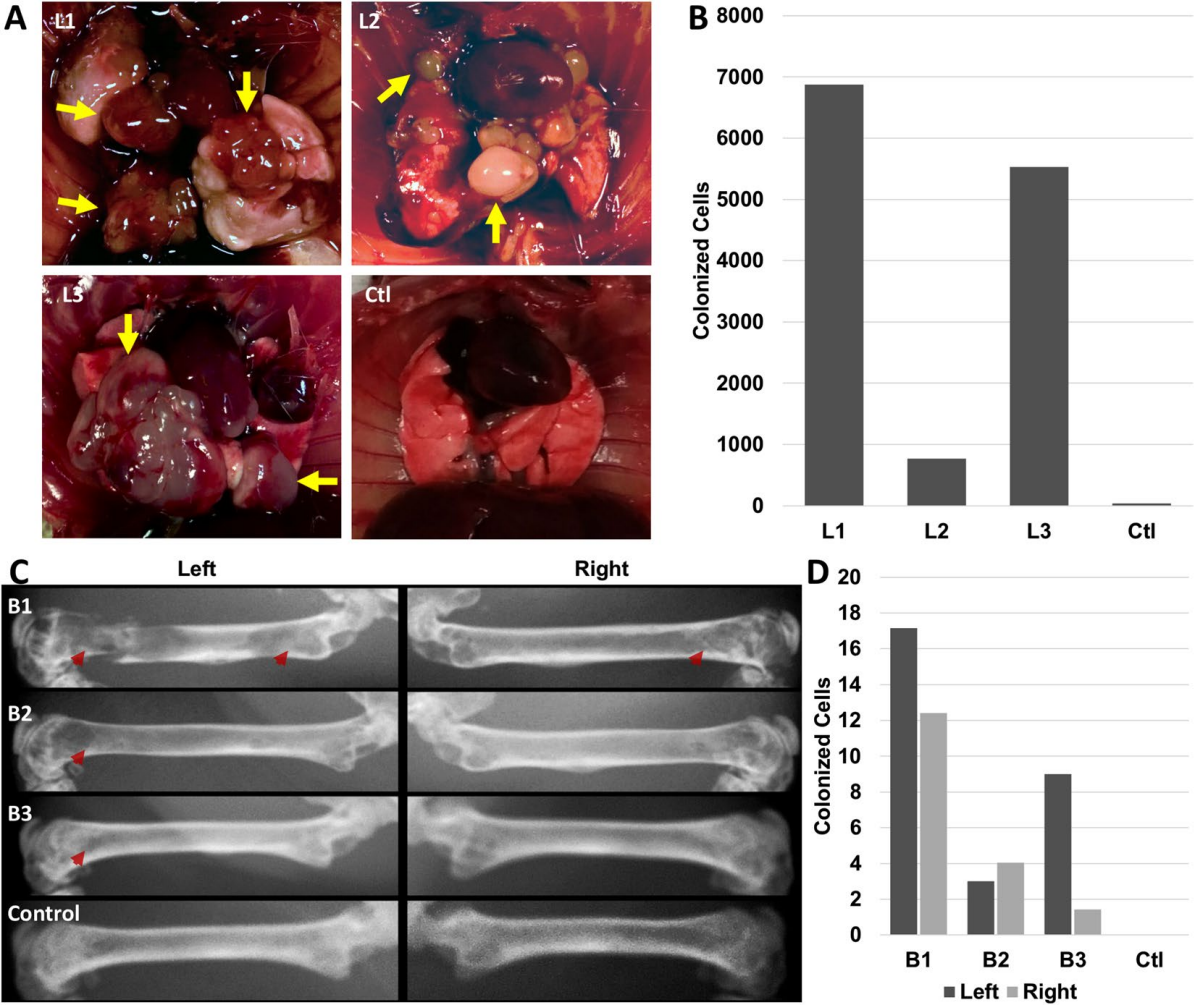
Tumorigenic and ^14^C activity in lung and bone metastasis. (**A**) Lung metastases of differing tumor burden 7-week following PC3 intracardiac injection from 4 mice and (**B**) quantification of colonized cells from AMS analysis of ^14^C signal in tissue DNA. (**C**) X-ray images of femurs from 4 animals 7-week post injection following PC3 intracardiac injection. Red arrows denote sites of osteolytic activity induced by cancer cells and (**D**) associated quantities of colonized cells in each femur.

In order to correlate ^14^C signal with the level of tumor burden, IC injected PC3 cells were analyzed for visible lung tumor formation at 7-weeks post injection followed by ^14^C signal quantification. ^14^C levels correlated with both the occurrence of tumors as well as relative tumor burden. Specifically, mouse lungs L1 and L3 exhibited extensive tumors and also yielded the largest levels of colonized cells. In contrast, DNA obtained from mouse L2 lungs produced detectable yet <20% the ^14^C signal compared to L1 and L3, which is consistent with the observed tumor burden. Additionally, control lung tissue with no metastases contained no detectable ^14^C levels (Fig. 5A,B).

IC injected cancer cells have also been shown to favor bone metastasis in several cancer types^33,34^. Because PC3 cells have been reported to produce osteolytic lesions upon metastasis to the bone, bone loss was assessed at 7-weeks post-IC injection *via* X-ray imaging and then compared to the ^14^C levels detected in the same bone derived DNA. The levels of colonized PC3 cells estimated *via* AMS quantification of ^14^C signal correlated with the extent of visible osteolytic lesions. The mouse (B1) with the most extensive visible osteolytic activity also had the highest number of colonized cells in both femurs (Fig. 5F,J). The mice with visible but reduced osteolytic activity (B2/B3) were also found to harbor fewer, but above control ^14^C levels (Fig. 5G–J). Interestingly, the quantity of tumor cells identified in bone metastatic tumors was very low, suggesting that a few cells are sufficient to colonize and seed metastatic bone tumors, in NSG mice.

## Discussion

The method presented herein is an extremely sensitive and informative tool for evaluating rare events of cancer cell colonization where we can detect one single cancer cell in a pool of 1 × 10^7^ cells. We have demonstrated the utility of Accelerator Mass Spectrometry (AMS) by evaluating metastatic patterns of cancer cell lines with different known metastatic profiles, and compared different delivery routes, in NSG mice. We were able to show, that less than 5% of human cancer cells delivered subcutaneously into NSG mice survive to form a tumor. Furthermore, we were able to show that metastatic tumors are initiated by very few cells, we found as low as 20 founder cells in kidney tumors, and few as 2 cells in bone tumors. Liver and lung tissues had the most tumor initiating cells, where >5000 cells persisted past 7-weeks, in liver tumors.

For the first time, we were able to track single founder cells to quantitatively study metastatic events in a variety of tissue types. While the assay presented here has provided novel insights into cell colonization, there are a few limitations of the model systems used. Cells delivered directly into the bloodstream do not have to extravasate from the primary tumor, therefore intravenous delivery of cancer cells does not reproduce all the events involved in metastasis. While spontaneous metastases are rare, the tumors evaluated had to extravasate the vasculature and infiltrate distant organs to colonize a secondary location, therefore they do partially mimic metastatic events. Future studies using this cell labeling assay may include orthotopic delivery of ^14^C-labeled cancer cells. Tracking metastatic events in which cells are required to undergo an epithelial to mesenchymal transition will help to parse out delivery method bias and will provide a more complete understanding of cancer cell colonization.

The utilization of AMS to track cancer cells *in vivo* represents a new application of a technology that can be further explored to study metastasis, and address key questions in cancer biology such as, why do some tumor cells remain dormant in tissues, while others rapidly form aggressive tumors. In particular the persistence of a few cells in the heart is intriguing. While we have optimized parameters such as concentration of ^14^C-thymidine in cell culture media during cancer cell labeling *in vitro* to allow for maximum ^14^C-thymidine retention once cells are removed from the labeling conditions, it remains to be determined whether similar approaches can be used on tumor cells isolated from primary tumors. If we can label and expand cancer cells obtained from biopsies, this approach may be further developed to evaluate the aggressiveness of tumors, and inform personalized treatments.

Additionally, we have optimized the length of time that cells are incubated with radiolabeled thymidine to allow for sufficient incorporation detectable at 12-week time points post labeling. We have also thoroughly investigated potential pitfalls of our assay to further substantiate our findings, such as metabolism of free ^14^C-thymidine at physiological concentration as well as possible reincorporation of ^14^C-thymidine *in vivo* into other dividing cells from dead cancer cells. Because the half-life of ^14^C is 5,730 years, this approach can be used to carry long term studies in pulse-chase type experiments, *in vivo*, without losing single cells resolution.

We have shown that the route of cancer cell administration influences where metastases occur, therefore xenograft studies need to be carefully assessed when trying to extrapolate data to patients. We found prostate cancer cells to preferentially target the kidney when introduced into the bloodstream through the tail vein but to colonize the bone when delivered through an intracardiac route of entry. This result is consistent with several published studies which support the idea that intracardiac inoculation is more likely to result in metastatic bone lesions *in vivo* compared to intravenous delivery^35,36^. Both visual osteolytic activity observed *via* x-ray imaging and individual cell quantification *via* AMS demonstrated that bone metastases were only achievable using an intracardiac delivery, despite the bone microenvironment being a well-known target of prostate cancer, presumably regardless of mode of entry^37^.

The number of ^14^C- labelled cells quantified in bone and lung tissues were correlative with the visual tumor burden and observable osteolytic activity when compared to control tissues, suggesting that quantification of ^14^C in the absence of visual tumors can be a reliable predictor of metastatic tumors. Even in the subcutaneous model, we showed that only 1–5% of implanted cells were present in the harvested tumors and label retention was still correlative with the initial founder cell population quantified using our assay and further suggesting that cell colonization is a rare event. This type of specificity for determining founder cell colonies is unprecedented in the literature. Even bioluminescence-based luciferase assays with current state of the art imaging platforms can only quantify normalized photon flux as a measure of labeled cell density in target organs, while we have shown that our assay quantifies individual cells based on direct incorporation of a radiolabel into cellular DNA^38^.

In practice, using AMS methodology to study metastasis is straightforward given proper equipment and institutional allowance of radioactivity use. The Accelerator Mass Spectrometer facility at LLNL has undergone years of research and development to be used in the capacity that it is today. Ongoing efforts aimed at developing a laser-based AMS instrument with commercial potential and requiring minimal lab space are underway. Until then, the AMS facility regularly collaborates with other research institutions that wish to utilize this technology making it possible for all investigators to benefit from this resource.

This novel methodology for understanding metastasis provides a new avenue for studying cancer in the future. We can now track specific cells *in vivo* to study their characteristics that allow them to colonize a target organ and form metastasis. Furthermore, we can couple xenograft experiments with drug treatment to evaluate efficacy and study drug resistance. Additionally, with the advent of single cell sequencing and other single cell characterization tools, we can gather genetic information about these radiolabeled cells that have metastasized to elucidate underlying genetic mechanisms. This AMS-based assay gives new hope for advancing the field of metastasis with detection of single cells in model organisms with an unprecedented level of specificity and accuracy.

## Material and Methods

### Cell culture and ^14^C-thymidine labeling

PC3 and LNCaP cell lines were obtained from ATCC (https://www.atcc.org/). PC3 cells were cultured in GIBCO® Dulbecco’s Modified Eagle Medium: Nutrient Mixture F-12 (DMEM/F-12) containing 10% dialyzed FBS and 1% pen/strep. LNCaP cells were cultured in GIBCO® RPMI 1640 containing 10% dialyzed FBS and 1% pen/strep.

PC3 cells were cultured in 0.55, 2.75, 5.5, or 11 nCi/mL [2-^14^C]-thymidine (Moravek Biochemicals) and ^14^C-thymidine DNA signal was quantified at days 2, 5, 7, 9 and 14 day for labeling optimization. Following optimization, cells were cultured in 11 nCi [2-^14^C]-thymidine in standard growth media for at least 7 days.

### Xenograft models

All animal experiments were approved by the Lawrence Livermore National Laboratory Institutional Animal Care and Use Committee and conform to the Guide for the care and use of Laboratory animals. NSG (*NOD.Cg-Prkdc*^*scid*^ *Il2rg*^*tm1Wjl*^*/SzJ*) mice received tumor cells through 3 specified routes of delivery. Intravenously tail vein (TV) administered mice received 100 µl of 1 × 10^7^cells/ml in PBS into the lateral tail vein. Intracardiac (IC) administered mice were first anesthetized with isofluorene then 100 μl of 1 × 10^7^cells/ml in PBS was injected into the left ventricle of the heart. Subcutaneous (SQ) administered mice received 100 μl of cell suspension in 1:1 Matrigel (Corning): PBS at various concentrations (1 × 10^3^ to 1 × 10^6^ cells per animal) below the skin of the right flank of the mouse. Tumor burden was assessed by measuring tumor length and width using digital calipers twice per week for up to 16 weeks. Tumor volume was estimated using the formula (length*width^2^)/2. Tumors were excised when they reached an estimated volume of 1 cm^3^.

### Tissue collection and DNA isolation

DNA was harvested from cells grown in culture and from collected tissues. Collected tissues were washed 3 times in PBS prior to tissue homogenization using a Bio-Gen Pro 200 homogenizer (PRO Scientific, Inc) in TEN buffer. ~25 mg of tissue (10 mg for spleen) was digested with Proteinase K and precipitated with isopropanol using commercially available silica-based columns (DNeasy Blood & Tissue Kit, Qiagen). Concentration of isolated DNA was quantified using a NanoDrop 2000 Spectrophotometer (ThermoFisher Scientific). Osteolytic activity was imaged via X-ray imaging using Bruker *In-Vivo* MS FX Pro. Organ photos were taken on a Leica MZ16FA fluorescence dissecting scope using Image Pro Plus software.

### Pharmacokinetics

To evaluate the pharmacokinetics of thymidine, 10-week old C57BL/6 male mice were administered a single intravenous dose of ^14^C-thymidine (700 pCi/animal) in sterile saline through an implanted jugular vein catheter. Following dose administration 1 drop of blood (approximately 20 µL) was collected from the catheter every 5 min up to 60 min and then at 2-, and 6- hours post dose, and deposited on a glass microfiber filter (Whatman, Maidstone, UK). Each filter paper was placed in a glass vial and stored at −80 °C until analysis by AMS.

### Liquid scintillation counting

The ^14^C-thymidine incorporated into the DNA of the cultured cells was quantified using liquid scintillation counting following DNA isolation and quantitation described above. Samples of DNA in water (250–1000 ng in 50 µL) were added to Universol scintillation cocktail (MP Biomedicals) and counted on a Perkin Elmer TriCarb 2910TR liquid scintillation analyzer.

### AMS measurements

Whole blood was analyzed neat by depositing drops on glass fiber filters. For DNA analysis, aliquots of DNA under 0.5 mg of carbon require addition of carrier carbon for efficient reduction of CO_2_ to elemental carbon and AMS analysis. Each DNA sample was dried in a quartz combustion vial (6 mm O.D. × 30 mm) using a vacuum centrifuge prior to the addition of carbon carrier. Each vial of DNA received 1-μL tributyrin (ICN Pharmaceuticals, Inc.; Costa Mesa, CA) added as carbon carrier (0.59 mg C) delivered with a volumetric capillary tube (Drummond Scientific Company, Broomall, PA) as described previously^21,39^. Each filter paper containing the collected blood was placed into a 6 × 55 mm quartz tube using disposable forceps. All samples were subsequently dried under vacuum centrifugation. The dried blood and DNA samples were then converted to graphite by a two-step process using published methods^40,41^. Briefly, the dried samples were oxidized to CO_2_ by heating at 900 °C for 4 h in the presence of copper oxide. The CO_2_ was then cryogenically transferred to a septa-sealed vial under vacuum and reduced to filamentous graphite on cobalt catalyst^40^. Graphite samples were packed into sample holders and carbon isotope ratios were measured on a National Electrostatics Corporation (Middleton, WI) compact 250 kV AMS spectrometer^25^. Typical AMS measurement times were 5–10 min/sample, with a counting precision (relative standard deviation, RSD) of 0.5% to 3% and a standard deviation among 3 to 10 measurements of 1% to 3%^24^. The ^14^C/^13^C ratios of the samples were normalized to measurements of four individually prepared isotopic standard samples prepared using the same method (IAEA C-6 also known as ANU sucrose) and converted to biological units as described in the “Metastasized Cell Colonization” section.

### Metastasized Cell Colonization

The natural contemporary concentration of ^14^C in living things is equivalent to one ^14^C atom in the complete genomes of 15 human cells. The radioactivity of purified DNA from the stock labeled cells is measured in triplicate by LSC for known masses of DNA measured by the nanodrop spectrophotometer. Given the mass of nuclear DNA in mouse cells is 6.2 pg, the number of ^14^C-TdR per cell in the labeled stock is calculated from the activity of the DNA and the DNA mass. The mass of DNA extracted from metastatic tumors, tissues from dosed animals, and undosed controls is then quantified with the spectrophotometer and analyzed by AMS using carrier carbon. Excess ^14^C above controls in the DNA of individual samples is then calculated to yield excess ^14^C/mass of DNA. The total mass of DNA extracted from a tissue/tumor sample is used to calculate the total excess ^14^C above contemporary control. The total excess ^14^C/^14^C per labeled dosing cell = number of labeled cells responsible for the tumor.

### HPLC Analysis

Ten-week-old C57Bl/6 male mice were administered a single intravenous dose of ^14^C-thymidine (700 pCi/animal) in sterile saline through an implanted jugular vein catheter. Animals were placed in metabolism cages and urine was collected for 24 h post dose. The collected urine was concentrated under vacuum and was analyzed by reversed-phase HPLC for thymidine. After centrifugation of each urine sample at 10,000 RPM for 5 min, 100 µl of the supernatant was directly injected into an Alliance HPLC system (Waters, Milford, MA) equipped with a 4 µm, 4.6 × 250 mm Synergi Max-RP 80 A column (Phenomenex, Torrance, CA), and monitored at 265 nm. Analytes were eluted at 0.5 ml/min initially at 100% solvent A. (Solvent A: 5% acetonitrile/95% 10 mM formic acid). This was followed by a gradient to 90% solvent B (solvent B: 90% acetonitrile/10% HPLC pure water) at 10 min., followed by a final gradient to 100% solvent A at 11 min. The solvent A concentration was maintained at 100% for 14 min. The column eluent was collected at 1 min intervals, and radioactivity was quantified by scintillation counting (PerkinElmer Packard).

### Statistical analysis

Data are presented as mean ± SEM. Significant differences were probed using Student t-test. Probability values < 0.05 were taken as significant. Probability values shown in figures correspond to *p < 0.05.
